# Cognitive Profiles and Atrophy Ratings on MRI in Senior Patients With Mild Cognitive Impairment

**DOI:** 10.3389/fnagi.2018.00384

**Published:** 2018-11-21

**Authors:** Marianne M. Flak, Haakon R. Hol, Susanne S. Hernes, Linda Chang, Thomas Ernst, Andreas Engvig, Knut Jørgen Bjuland, Bengt-Ove Madsen, Elisabeth M. S. Lindland, Anne-Brita Knapskog, Ingun D. Ulstein, Trine E. E. Lona, Jon Skranes, Gro C. C. Løhaugen

**Affiliations:** ^1^Department of Clinical and Molecular Medicine, Norwegian University of Science and Technology, Trondheim, Norway; ^2^Department of Pediatrics, Sørlandet Hospital HF, Arendal, Norway; ^3^Department of Radiology, Sørlandet Hospital HF, Arendal, Norway; ^4^Department of Clinical Science, University of Bergen, Bergen, Norway; ^5^The Memory Clinic Geriatric Unit, Department of Medicine, Sørlandet Hospital, Arendal, Norway; ^6^Department of Diagnostic Radiology and Nuclear Medicine, and Department of Neurology, University of Maryland School of Medicine, Baltimore, MD, United States; ^7^Department of Neurology, Johns Hopkins University School of Medicine, Baltimore, MD, United States; ^8^Department of Medicine, John A. Burns School of Medicine, University of Hawaii at Manoa, Honolulu, HI, United States; ^9^Department of Medicine, Diakonhjemmet Hospital, Oslo, Norway; ^10^Department of Research, Sørlandet Hospital, Arendal, Norway; ^11^Department of Radiology and Nuclear Medicine, Oslo University Hospital, Oslo, Norway; ^12^Institute of Clinical Medicine, University of Oslo, Oslo, Norway; ^13^Department of Geriatric Medicine, The Memory Clinic, Oslo University Hospital, Oslo, Norway; ^14^Department of Psychiatry, Age Psychiatry, The Hospital of Telemark, Skien, Norway

**Keywords:** MCI, intelligence, memory clinic patients, cognitive dysfunction, brain pathology, structural magnetic resonance imaging, neuropsychological functioning, neuropsychological tests

## Abstract

In this cross-sectional study, we sought to describe cognitive and neuroimaging profiles of Memory clinic patients with Mild Cognitive Impairment (MCI). 51 MCI patients and 51 controls, matched on age, sex, and socio-economic status (SES), were assessed with an extensive neuropsychological test battery that included a measure of intelligence (General Ability Index, “GAI,” from WAIS-IV), and structural magnetic resonance imaging (MRI). MCI subtypes were determined after inclusion, and z-scores normalized to our control group were generated for each cognitive domain in each MCI participant. MR-images were scored by visual rating scales. MCI patients performed significantly worse than controls on 23 of 31 cognitive measures (Bonferroni corrected *p* = 0.001), and on 8 of 31 measures after covarying for intelligence (GAI). Compared to nonamnestic MCI patients, amnestic MCI patients had lower test results in 13 of 31 measures, and 5 of 31 measures after co-varying for GAI. Compared to controls, the MCI patients had greater atrophy on Schelten's Medial temporal lobe atrophy score (MTA), especially in those with amnestic MCI. The only structure-function correlation that remained significant after correction for multiple comparisons was the MTA—long delay recall domain. Intelligence operationalized as GAI appears to be an important moderator of the neuropsychological outcomes. Atrophy of the medial temporal lobe, based on MTA scores, may be a sensitive biomarker for the functional episodic memory deficits associated with MCI.

## Introduction

The term “Mild Cognitive Impairment” (MCI) is currently understood as a clinical condition characterized by reduction in memory and/or other cognitive processes not severe enough to meet the criteria for dementia, but more pronounced than the cognitive decline associated with normal aging (Reisberg and Ferris, 1982; Petersen et al., 1997, 1999; Reisberg et al., 2008; Geda and Nedelska, 2012). According to the Petersen criteria, MCI is operationalized as objective impairment on neuropsychological tests, in combination with intact general cognitive functioning and activities of daily living (Petersen et al., 1999). Initially, MCI was constructed as a transition stage between nonimpaired cognitive aging and Alzheimer's disease. Since then the initial focus on memory impairment in the MCI criteria has expanded. The 2004 revised criteria further classified MCI into “amnestic” and “nonamnestic” subtypes (Petersen, 2004; Winblad et al., 2004). When a diagnosis of MCI is established, its subtype is defined by the results of the individuals neuropsychological profile. In amnestic MCI, the profiles indicates deficit within the memory domain. Conversely, nonamnestic MCI indicates intact memory, but impaired function in other domains for instance working memory or executive function (Petersen et al., 1997, 1999; Collie and Maruff, 2002; Collie et al., 2002; Boeve et al., 2003; Lopez et al., 2003, 2006; Winblad et al., 2004; Petersen and Knopman, 2006; Petersen and Negash, 2008). There is a lack of studies that describe cognitive profiles of amnestic and nonamnestic MCI patients diagnosed in a memory clinic by national guidelines. Knowledge about the individuals neuropsychological profiles has clinical and prognostic value, since patients with amnestic MCI, especially with multi-domain cognitive deficits, are more likely to progress more rapidly to dementia (Arnáiz et al., 2001; Bozoki et al., 2001; Tuokko et al., 2003; Luis et al., 2004). In addition to prognosis, knowledge of functional strengths and weaknesses are important for potential treatment, interventions and guidance for caregivers (Ten Kate et al., 2017). New clinical criteria for the diagnosis of Alzheimer's disease recommend the use of biomarkers (e.g., structural brain imaging, and cerebrospinal fluid analyses) also in patients with MCI (Dubois et al., 2007; Albert et al., 2011; Jack et al., 2011; McKhann et al., 2011). Clinical markers of MCI include cognitive function assessed by neuropsychological tests as described above, and signs of structural brain pathology on magnetic resonance imaging (MRI) (Petersen and Negash, 2008; Jak et al., 2009).

Rog and Fink (2013) recommended that cognitive assessment in MCI should include all major neuropsychological domains (i.e., attention, working memory, visual and verbal learning and memory, processing speed, and executive function) and ideally also an estimate of general cognitive ability. The aging brains' ability to tolerate structural damage relates to the resilience, or “reserve,” of the brain (Stern, 2013). General cognitive ability is an estimate of an individual's ability prior to the onset of a pathological process, a premorbid function. The notion of “cognitive reserve” as a mediator of structure-function relationship between brain and cognition in aging is well-established (Katzman et al., 1988; Stern, 2002; Robertson, 2013). Intelligence can be considered a proxy for cognitive reserve (Richards and Deary, 2005; Osone et al., 2015). Intelligence is usually assessed with structured psychometric tests. One of the most widely used test batteries worldwide is the Wechsler Adult Intelligence scale—fourth edition (WAIS-IV). WAIS-IV produces a composite score that represents general intellectual ability: The Full Scale Intelligence Quotient (IQ), which includes the following indexes: Verbal comprehension, Perceptual reasoning, Working Memory, and Processing speed. However, a problem of using Full Scale IQ as a measure of cognitive reserve in MCI patients is that this composite score also includes the domains of Working Memory and Processing speed that are vulnerable to aging in general (Schaie, 1994), and MCI in particular (Salthouse and Meinz, 1995). This may result in lower scores on Full Scale IQ in patients with MCI than nonimpaired individuals, without the intellectual ability *per se* being reduced. WAIS-IV also include a composite IQ score that consist only of the Verbal comprehension subtests and Perceptual reasoning subtests: General Ability Index (GAI), resulting in a measure of general ability that is not sensitive to the influence of the working memory- and processing speed abilities (Tulsky et al., 2001). Hence, the GAI is a measure of IQ not including subtests of cognitive proficiency, and may therefore be a better measure of intellectual ability or cognitive reserve than Full scale IQ in a clinical group of patients with MCI.

Most research in this field has been performed with functional MRI, since fMRI incorporates both structural localization and to some degree a measure of function in one examination. However, the most readily available methods in clinical use for evaluating brain structure-function relationships are neuropsychological tests and semi-quantitative scoring systems based on visual reading of MRI. Several radiological scoring systems for clinically evaluating brain pathology markers related to dementia exist (Ferreira et al., 2015, 2016; Rhodius-Meester et al., 2017). Temporal lobe atrophy is mainly evaluated by the medial temporal lobe atrophy score (MTA) (Scheltens et al., 1995). The amnestic MCI subtype, often regarded as prodromal to Alzheimer's disease (AD), is typically associated with hippocampal atrophy assessable by the MTA score, and memory impairment (Petersen and Morris, 2005). Other evaluation tools like the Posterior Atrophy (PA) score (Koedam et al., 2011) and the Global Cortical Atrophy Frontal (GCA-F) sub score (Pasquier et al., 1996; Ferreira et al., 2016) are potential biomarkers for early onset AD, atypical AD, and nonAD dementia (Ferreira et al., 2017). White matter hyperintensities (WMH), depending on lesion frequency and location, were also associated with cognitive decline (Overdorp et al., 2014; Prins and Scheltens, 2015). A clinical scoring system for how extensive the WHI are is the Fazekas score. This WMH scoring system is recommended for cognitive impairment research by the Imaging Cognitive Impairment Network group, together with the radiological atrophy scores (Wahlund et al., 2017). Broad spectra of individual anatomical differences, cognitive reserve, and varieties in brain structural changes exist due to normal aging compared to that of neurodegenerative diseases. Therefore, separating patients with MCI from cognitively nonimpaired individuals based exclusively on structural MRI is difficult (Gómez-Sancho et al., 2018). More research in this field have been recommend by the Geneva Task Force for the Roadmap of Alzheimer's Biomarkers (Ten Kate et al., 2017).

In the present cross-sectional descriptive study, we aimed to investigate and describe the differences between patients with MCI to nonimpaired individuals on a comprehensive neuropsychological test battery that included an estimate of cognitive reserve (GAI) and visual radiological scoring systems. Further, we aimed to investigate if the degree of brain pathology identified by the clinical visual scoring systems on MRI is correlated with the scores on neuropsychological domains of attention, working memory, visual and verbal episodic learning and memory, processing speed, and executive function in patients with MCI (Rog and Fink, 2013). We hypothesized that the MCI patients would have inferior scores on several cognitive domains, but that GAI would moderate group differences. We also hypothesized that greater degree of brain pathology would be found in those with lower scores on the cognitive domains.

## Materials and methods

### Participants

The study was approved by the Norwegian Regional Committee for medical and health research ethics, South-Eastern Health region (no: 2013/410) and by the Department of Research at each collaborating hospital. Fifty one patients diagnosed with MCI were recruited from Memory clinics at four hospitals in the South-Eastern Health Region in Norway (Sørlandet Hospital Arendal, Telemark Hospital, Oslo University Hospital, and Diakonhjemmet Hospital). Eligible patients with MCI were invited to participate between August 2013 and December 2016. The study participants were assessed with neuropsychological tests, questionnaires for risk factors ascertainment and MRI of the brain as specified by the Norwegian national guidelines (NorCog) and the diagnoses of MCI were made in accordance with the Petersen/Winblad criteria of MCI (Petersen, 2004; Winblad et al., 2004). Classification of MCI subtypes is not done routinely in the Memory clinics. Therefore, this classification was performed after inclusion, according to the patients cognitive profiles (cut-off at one neuropsychological test impaired per domain, >1.5 SD below age- and gender-appropriate norms). The study neuropsychologist categorized the MCI participants into amnestic and nonamnestic MCI based on their scores on the neuropsychological tests in the baseline assessment. Scores more than −1.5 SD from mean compared to norms on the tasks within the verbal and/or visual episodic memory domain were classified as amnestic MCI. Normal scores in memory domains combined with scores more than −1.5 standard deviation from the mean in one or more of the other domains assessed, resulted in categorization as nonamnestic MCI (Petersen et al., 1999, 2001; Winblad et al., 2004).

A control group of 51 volunteers was recruited through Sørlandet hospital's website, local newspapers, and radio. They were matched to the MCI group by sex, age, and socioeconomic status (SES). SES was calculated using Hollingshead's index of education and occupational position, scaled from 1 (low) to 5 (high) (Hollingshead and Redlich, 1958). The participants underwent neuropsychological assessment and brain MRI. Exclusion criteria included head trauma with post-traumatic loss of consciousness during the lifespan, photosensitive epilepsy, or person unsuitable for MRI because of inserted metal or severe claustrophobia.

See Tables 1, 2 for clinical characteristics of the groups.

**Table 1:** Clinical characteristics and cognitive scores in patients with MCI (*n* = 51) and controls (*n* = 51).

	**MCI *n* = 51**	**Controls *n* = 51**
	**Mean (SD or range)**	**Mean (SD or range)**	**Exact** ***p*****-value**
Age at assessment, years	66 (51–80)	66 (53–81)	0.849
Males/females	35/16	35/16
Education, years	13 (8–20)	14 (10–19)	0.198
Socioeconomic status	3.4 (1.1)	3.7 (1.0)	0.339
Full Scale Intelligence Quotient WAIS-IV	96 (15)	110 (12)	< 0.0001
General Ability Index (WAIS-IV)	100 (16)	114 (13)	< 0.0001
Verbal comprehension Index (WAIS-IV)	100 (14)	110 (12)	< 0.0001
Perceptual organization Index (WAIS-IV)	101 (17)	113 (13)	< 0.0001
Working memory Index (WAIS-IV)	92 (13)	106 (17)	< 0.0001
Processing speed Index (WAIS-IV)	94 (14)	104 (18)	< 0.0001

*Mann-Whitney U-tests for nonparametric variables. The significance level is 0.05*.

MCI, Mild Cognitive Impairment; SD, Standard Deviation; WAIS-IV, Wechsler Adult Intelligence Scale, 4th edition

**Table 2:** Clinical characteristics and cognitive scores in patients with amnestic (*n* = 35) and nonamnestic (*n* = 16) MCI.

	**aMCI *n* = 35**	**naMCI *n* = 16**
	**Mean (sd or range)**	**Mean (sd or range)**	**Exact** ***p*****-value**
Age at assessment, years	66 (53–80)	65 (51–80)	0.707
Males/females	24/11	11/5
Education, years	13 (8–20)	15 (12–18)	0.026
Socioeconomic status	3.2 (1.2)	3.9 (1.0)	0.030
Full IQ WAIS-IV	91 (13)	107 (10)	< 0.0001
General ability Index (WAIS-IV)	95 (15)	112 (12)	< 0.0001
Verbal comprehension Index (WAIS-IV)	95 (12)	111 (12)	< 0.0001
Perceptual organization Index (WAIS-IV)	97 (17)	110 (13)	0.010
Working memory Index (WAIS-IV)	87 (11)	102 (11)	< 0.0001
Processing speed Index (WAIS-IV)	90 (14)	102 (11)	< 0.008

*Mann-Whitney U-tests for nonparametric variables. The significance level is 0.05. Abbreviations: MCI, Mild Cognitive Impairment; SD, Standard Deviation; WAIS-IV, Wechsler Adult Intelligence Scale, 4th edition*.

### Neuropsychological assessment

A neuropsychological test battery assessed the following cognitive domains: intelligence, attention, working memory, processing speed, visual episodic learning/short delay recall, visual episodic memory/long delay recall, verbal episodic learning/short delay recall, verbal episodic memory/long delay recall and executive functions. Standardized, internationally renowned neuropsychological tests were applied. All tests were administered in a fixed order by the same clinical neuropsychologist (MMF) to all study participants. The WAIS-IV is considered a valid and reliable battery for intelligence testing in an adult population. It generates two general measures of cognitive function Full scale IQ and GAI (Strauss et al., 2006; Wechsler, 2008; Sattler and Ryan, 2009; Lezak, 2013). The GAI was chosen as a measure of intelligence in our study. The neuropsychological tests are listed in Table 3.

**Table 3:** Assessed cognitive domains and neuropsychological tests.

**Cognitive domains**	**Tests**
Intelligence (IQ)	WAIS-IV (General Ability Index/GAI)
Attention domain	WAIS-IV Digit Span forward, WMS-III Spatial Span forward, CVLT-II Trial 1, CVLT-II Trial B
Working memory domain	WMS-IV Digit Span backward, WMS-III Spatial Span backward, WMS-III Letter-Number Sequencing
Processing speed domain	WAIS-IV Coding, WAIS-IV Symbol search, D-KEFS Color Word Interference Test 1 color naming, D-KEFS Color Word Interference Test 2 Word reading
Visual episodic learning/short delay recall domain	RCFT Immediate recall, WMS-III Faces I
Visual episodic memory/long delay recall domain	RCFT Delayed Recall, WMS-III Faces II Delayed recall
Verbal episodic learning/short delay recall	WMS-III Logical Memory I, CVLT-II Total learning, CVLT-II Short Delay Free Recall
Verbal episodic memory/ long delay recall	Logical memory II Delayed recall, CVLT-II Long delay free recall, CVLT Total hits
Executive functions	RCFT, D-KEFS Color Word Interference Test 3 Inhibition, D-KEFS Color Word Interference test 4 Inhibit/Switching, D-KEFS Verbal Fluency Test Letter fluency, D-KEFS Verbal Fluency Test Category fluency, D-KEFS Verbal Fluency Test Category switching

*WAIS-IV, Wechsler Adult Intelligence Scale 4.ed, WMS-III, Wechsler Memory Scale 3.ed, D-KEFS, Delis-Kaplan Executive Function System, RCFT, Reys Complex Figure Test, CVLT-II, California Verbal Learning Test 2.ed*.

## Cerebral MRI

### Data acquisition

Images were acquired from three different 1.5 Tesla Siemens Aera MR Systems. Study participants were scanned with a standardized protocol containing volumetric T1-weighted magnetization-prepared rapid gradient echo (MP-RAGE) and fluid attenuation inversion recovery (FLAIR) sequences. Following a pilot scan, two three-dimensional (3D) MP-RAGE scans (sagittal, echo time 3.47 ms, repetition time 2,400 ms, TI 1,000 ms, flip angle 8 degrees, 1.2 mm resolution covering the whole brain) and a 3D-T2 weighted fluid attenuated inversion recovery (FLAIR) image (sagittal, echo time 335 ms, repetition time 5,000 ms, TI 1,800 ms, turbo factor 242, 1.2 mm resolution covering the whole brain) were performed. Total scan time was 30 min.

### Scoring systems and data analysis

Visual radiological scoring systems were used to assess brain pathology in the MCI patients and controls. These scales included Scheltens Medial temporal lobe atrophy (MTA) score, the Fazekas's scale for WMH, Global cortical atrophy—frontal (GCA-f) sub score and PA score (Table 4).

**Table 4:** Radiological scoring systems, range, region and age cut off.

**Radiological scoring system**	**Anatomical region/structure**	**Range**	**Pathological age cut off**
MTA	Medial temporal lobes/hippocampus	0–4	1.0 ≤ 64 years 1.5 ≥ 65–74 years ≥ 2 >75 y.
PA	Parietal lobes	0–3	≥2 for all ages.
GCA-F	Frontal lobes	0–3	≥1.5 for all ages.
FAZEKAS	White matter	0–3	1 for all groups.

*MTA, Scheltens Medial temporal lobe atrophy score, PA, Koedam's posterior atrophy score. GCA-F, Passchier's Global cortical atrophy score—frontal subscore. Fazekas, Fazekas white matter hyperintensity (WHI) score*.

We evaluated the MTA, PA, and GCA-F scores on the T1w images, and the Fazeka's score on the FLAIR images. For the visual rating, two experienced radiologists viewed the images independently at separate locations. Both radiologists were blinded toward group allocation. Reference images for all scores were provided for both radiologists as suggested by Harper et al. (2015). A consensus rating was held if a disagreement existed. For all scores except the Fazekas and PA scores, both brain hemispheres were scored and a mean score was calculated (Schoonenboom et al., 2008; Ferreira et al., 2016). A mean score was calculated based on both brain hemispheres for the MTA and GCA-F (Schoonenboom et al., 2008; Ferreira et al., 2016). The MTA score cut-offs were set at ≥1.0 for persons under 65, at ≥1.5 for persons between 66 and 74 years of age, and at ≥2 for those ≥ 75 years (Ferreira et al., 2017; Rhodius-Meester et al., 2017). The MTA score ranges from 0 to 4 (from 0 = no atrophy to 4 = most severe atrophy), which describes the relative size of the hippocampus at a fixed position on T1 images. GCA-F utilized a cutoff at ≥1.5 for all ages (Rhodius-Meester et al., 2017). The GCA-F describes the atrophy severity of the frontal lobe, and scores range from 0 to 3 (0 = no atrophy, 1 = mild atrophy, 2 = moderate atrophy, 3 = severe atrophy). The PA scoring system (PA) also ranges from 0 to 3 (0 = no atrophy, 1 = mild, 2 = moderate, 3 = severe atrophy) and was used with the original age cutoff for PA ≥2 (Koedam et al., 2011). Fazekas scores categorize the nonspecific white matter hyperintensity load. The scores range from 1 to 3 (from absent to higher white matter lesion load depending on the location of the hyperintensities, see footnote in **Table 7**); a score >1 was considered pathological for all age groups (Fazekas et al., 1987) For all radiological scoring systems, scores above the set cut-off values were considered pathological.

### Statistical analysis

Statistical analyses were performed using IBM SPSS statistics, version 23.0. The Mann-Whitney *U*-tests for nonparametric variables were used to explore group differences in demographic variables (see Tables 1, 2). Multivariate analyses of variance within the General linear model, was used for between-group analyses (MCI patients and controls). Covarying for sex, age, SES, and years of education in the statistical model did not change the significance levels or frequency. The only covariate in the mixed model was the GAI.

In order to compare cognitive performance across groups and for the different domains, a z-score was calculated for each domain in each participant, based on the difference from the median score of the neuropsychological test scores in the control group divided by the standard deviation of the control group ($z\;=\;\frac{x-median_{controls}}{sd}$) (Yonelinas et al., 2002). An alpha level < 0.001 was considered statistically significant after Bonferroni-adjustment for multiple comparisons of the 31 neuropsychological outcomes. In order to reduce the number of variables in the structure-function correlation analyses, the neuropsychological z-scores were clustered into cognitive domains (Rog and Fink, 2013). Each neuropsychological domain score was correlated with each radiological score in linear regression analysis. The linear regression analysis was performed for each radiological score separately as a part of a hierarchic regression analyses. Age and sex were added as covariates.

The z-score domains were analyzed with and without GAI as a covariate in the model. For the MTA, PA, GCA-F, and Fazekas scores, two-tailed independent sample *T*-tests were applied to investigate possible differences in radiological scores between the MCI and the control group, and between the amnestic and nonamnestic MCI groups. For prevalence calculations, the radiological scores were dichotomized, according to their age cut-off. A Chi Square test was applied to investigate associations between groups and the dichotomized scores. We used linear regressions to model the relationship between the cognitive domains and the radiological scores. Statistical significance for these analyses was set to a *p* < 0.05.

## Results

Table 1 displays clinical characteristics of the study participants. Age, gender distribution, education, and SES showed no significant group differences. Conversely, there were significant group differences for the results on the WAIS-IV including the intelligence indices Full Scale IQ and GAI.

### MCI subtypes

In the MCI group, 35 participants were classified into the amnestic subtype, while 16 participants were classified into the nonamnestic subtype. Table 2 describes the characteristics of the two MCI subtypes. Statistically significant differences between the groups were found on all test variables with lowest scores in the amnestic subtype group.

### Neuropsychological test results

Compared to the controls, the MCI group showed lower performance on 23 out of 31 of the cognitive outcomes (Bonferroni adjusted *p* < 0.001). However, fewer results, 8 out of 31 outcomes, remained significantly different between the two groups when the GAI was included as a covariate to adjust for premorbid cognitive functioning. Specifically, tasks assessing the verbal episodic learning domain and verbal episodic memory domain (California Verbal Learning Test-II, and Logical memory I and II) remained significantly different between the groups. Furthermore, significant group differences remained on a test of executive function (Verbal Fluency Test Category Fluency) (Table 5).

**Table 5:** Neuropsychological test results in patients with MCI (*n* = 51) compared to matched controls (*n* = 51).

**Cognitive domain**	**Subtask**	**MCI (*n* = 51)**	**Controls (*n* = 51)**	***p*-value unadjusted**	*p*-value adjusted^*^
Attention	WAIS-IV Digit Span forward (items correct)	8.0 (1.7)	9.4 (2.4)	0.003	0.250
	WAIS-IV Digit Span forward, longest number of digits	5.4 (1.0)	6.3 (1.3)	<**0.0001**	0.101
	WMS-III Spatial Span forward (items correct)	6.4 (2.0)	7.3 (1.9)	0.025	0.624
	WMS-III Spatial Span forward, longest number of items	4.6 (1.2)	5.2 (1.0)	0.005	0.203
	CVLT-II Trial 1 (number of correct words)	4.0 (1.6)	5.8 (1.5)	<**0.0001**	<**0.0001**
	CVLT-II Trial B interference (number of correct words)	3.7 (1.6)	5.4 (2.1)	<**0.0001**	**0.001**
Working memory	WAIS-IV Digit Span backward (items correct)	6.7 (2.0)	8.0 (2.6)	0.011	0.645
	WAIS-IV Digit Span backwards, longest number of digits	3.8 (1.0)	4.5 (1.4)	0.013	0.510
	WMS-III Spatial Span backward (items correct)	5.8 (2.0)	7.6 (1.4)	<**0.0001**	0.002
	WMS-III Spatial Span backward, longest number of items	4.3 (1.0)	5.2 (0.8)	<**0.0001**	0.004
	WMS-III Letter-Number Sequencing (correct items)	7.4 (2.6)	10.1 (2.6)	<**0.0001**	0.008
Processing speed	WAIS-IV Coding (WAIS-IV) (number of items)	43.0 (14.4)	55.0 (13.4)	<**0.0001**	0.038
	WAIS-IV Symbol search (WAIS-IV) number of items	22.0 (7.5)	27.4 (8,0)	0.002	0.190
	D-KEFS Color Word Interference Test 1 color naming (seconds to complete)	40.0 (14.5)	31.6 (6.1)	**0.001**	0.051
	D-KEFS Color Word Interference Test 2 word reading (seconds to complete)	30.4 (15.1)	22.6 (4.2)	**0.001**	0.094
Visual episodic learning/ short delay recall	Rey Complex Figure Test Immediate recall (items remembered)	13.0 (8.0)	19.0 (6.0)	<**0.0001**	0.011
	WMS-III Faces I (items correct)	34.2 (4.7)	36.4 (4.1)	0.035	0.088
Visual episodic memory, long delay recall	Rey Complex Figure Test Delayed Recall (items correct)	12.0 (8.0)	18.5 (5.1)	<**0.0001**	0.002
	WMS-III Faces II Delayed recall (items correct)	32.3 (4.6)	36.2 (5.7)	<**0.0001**	0.006
Verbal episodic learning/ short delay recall	WMS-III Logical Memory I(number of items)	27.9 (12.4)	40.5 (8.4)	<**0.0001**	<**0.0001**
	CVLT-II Total learning (number of correct words)	33.5 (11.0)	47.8 (10.6)	<**0.0001**	<**0.0001**
	CVLT-II Short Delay Free Recall (number of word)	5.5 (4.0)	10.2 (3.2)	<**0.0001**	<**0.0001**
Verbal episodic memory, long delay recall	Logical memory II Delayed recall (number of items)	13.2 (9.4)	25.3 (6.2)	<**0.0001**	<**0.0001**
	CVLT-II Long delay free recall (number of words remembered)	5.2 (4.5)	10.0 (3.5)	<**0.0001**	<**0.0001**
	CVLT Total hits (words recognized)	12.7 (4.7)	15.1 (1.4)	**0.001**	0.053
Executive functions	Rey Complex figure Copy trial (number of items)	31.0 (6.0)	34.5 (2.0)	<**0.0001**	0.090
	D-KEFS Color Word Interference Test 3 Inhibition (seconds to complete)	86.0 (35.6)	60.9 (15.0)	<**0.0001**	0.019
	D-KEFS Color Word Interference test 4 Inhibit/Switching (seconds to complete)	101.0 (38.5)	70.1 (18.0)	<**0.0001**	0.002
	D-KEFS Verbal Fluency Test Letter fluency (number of words)	41.0 (17.7)	45.8 (11.8)	0.087	0.967
	D-KEFS Verbal Fluency Test Category fluency (number of words)	34.6 (11.5)	46.9 (11.1)	<**0.0001**	<**0.0001**
	D-KEFS Verbal Fluency Test Category switching (number correct)	11.2 (3.7)	14.0 (3.5)	**0.001**	0.038

*Mann Whitney U-test for nonparametric variables*.

* *General linear model, multivariate, with General Ability Index as covariate. Bonferroni correction for multiple comparisons: significant p-values (p < 0.001) in bold*.

*MCI, Mild Cognitive Impairment, CVLT-II, California Verbal Learning Test 2rd edition, WAIS-IV, Wechsler Adult Intelligence Scale 4th edition, WMS-III, Wechsler Memory Scale 3rd edition, D-KEFS, Delis Kaplan Executive Function System*.

**Figure 1:**
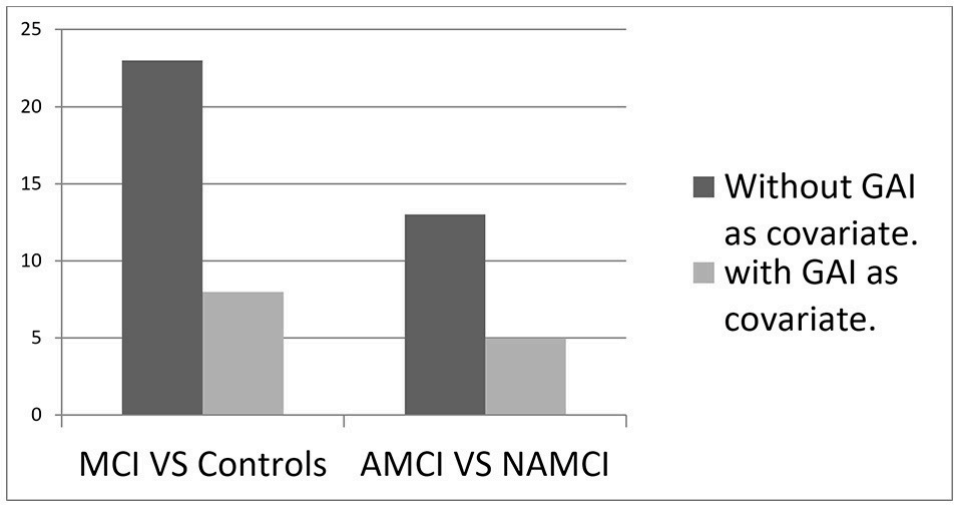
The number of significantly inferior test (*p* < 0.001) in MCI compared to controls or in amnestic MCI (AMCI) compared to nonamnestic MCI (NAMCI).

Analyses of group differences between the amnestic and nonamnestic MCI subtypes revealed significantly inferior scores in the amnestic group on 13 out of 31 cognitive outcomes. In the multivariate model, with GAI as covariate, only eight measures remained significantly different between groups (Table 6). See Figure 1 for visual display of data.

**Table 6:** Neuropsychological test results in patients with amnestic MCI (aMCI) (*n* = 35) and nonamnestic MCI (naMCI) (*n* = 16).

**Cognitive domain**	**Subtask**	**aMCI *n* = 35**	**naMCI *n* = 16**	***p*-value unadjusted**	*p*-value Adjusted^*^
Attention	WAIS-IV Digit Span forward (items correct)	7.5 (1.4)	9.2 (1.7)	**0.001**	0.028
	WAIS-IV Digit Span forward, longest number of digits	5.1 (0.8)	6.2 (0.8)	<**0.0001**	0.012
	WMS-III Spatial Span forward (items correct)	5.9 (1.9)	7.4 (1.8)	0.017	0.330
	WMS-III Spatial Span forward, longest number of items	4.4 (1.1)	5.1 (1.2)	0.054	0.203
	CVLT-II Trial 1 (number of correct words)	3.6 (1.5)	4.8 (1.6)	0.009	0.088
	CVLT-II Trial B interference (number of correct words)	3.4 (1.4)	4.3 (2.1)	0.073	0.375
Working memory	WAIS-IV Digit Span backward (items correct)	6.1 (1.7)	8.0 (1.9)	0.010	0.091
	WAIS-IV Digit Span backwards, longest number of digits	3.5 (0.9)	4.4 (1.0)	0.005	0.115
	WMS-III Spatial Span backward (items correct)	5.3 (1.7)	6.9 (2.0)	<**0.0001**	0.298
	WMS-III Spatial Span backward, longest number of items	4.1 (1.0)	4.9 (1.0)	0.017	0.004
	WMS-III Letter-Number Sequencing (correct items)	6.5 (2.2)	9.5 (2.0)	<**0.0001**	0.028
Processing speed	WAIS-IV Coding (number of items)	38.2 (13.4)	53.2 (11.0)	<**0.0001**	0.083
	WAIS-IV Symbol search (number of items)	21.0 (7.8)	24.5 (6.3)	0.108	0.437
	D-KEFS Color Word Interference Test 1 color naming (seconds to complete)	43.9 (15.5)	31.6 (6.9)	0.002	0.168
	D-KEFS Color Word Interference Test 2 word reading (seconds to complete)	33.5 (17.0)	23.7 (6.1)	0.020	0.505
Visual episodic memory, short delay recall	Rey Complex Figure Test Immediate recall (items remembered)	9.9 (6.8)	19.9 (6.2)	<**0.0001**	**0.001**
	WMS-III Faces I (items correct)	33.4 (4.9)	35.9 (3.7)	0.061	0.239
Visual episodic memory, long delay recall	Rey Complex Figure Test Delayed Recall (items correct)	8.9 (6.4)	19.0 (6.4)	<**0.0001**	<**0.0001**
	WMS-III Faces II Delayed recall (items correct)	31.6 (5.1)	33.9 (2.9)	0.101	0.229
Verbal episodic memory, short delay recall	WMS-III Logical Memory I (number of items)	24.6 (11.9)	35.2 (10.6)	0.005	0.049
	CVLT-II Total learning (number of correct words)	29.2 (9.3)	42.9 (8.7)	<**0.0001**	**0.001**
	CVLT-II Short Delay Free Recall (number of word)	3.8 (3.2)	9.3 (2.5)	<**0.0001**	<**0.0001**
Verbal episodic memory, long delay recall	Logical memory II Delayed recall (number of items)	10.4 (8.2)	19.3 (9.2)	0.003	0.017
	CVLT-II Long delay free recall (number of words remembered)	3.5 (3.7)	8.9 (4.0)	<**0.0001**	<**0.0001**
	CVLT-II Total hits (words recognized)	11.7 (5.3)	14.8 (1.7)	0.016	0.441
Executive functions	Rey Complex figure Copy trial (number of items)	30.3 (6.6)	33.7 (3.1)	<**0.0001**	0.847
	D-KEFS Color Word Interference Test 3 Inhibition (seconds to complete)	97.5 (36.2)	60.6 (16.0)	**0.001**	0.066
	D-KEFS Color Word Interference test 4 Inhibit/Switching (seconds to complete)	112.0 (38.3)	76.1 (25.4)	<**0.0001**	0.079
	D-KEFS Verbal Fluency Test Letter fluency (number of words)	36.6 (14.1)	51.0 (15)	0.004	0.026
	D-KEFS Verbal Fluency Test Category fluency (number of words)	32.0 (10.1)	40.1 (11.1)	0.009	0.066
	D-KEFS Verbal Fluency Test Category switching (number correct)	10.3 (3.7)	13.5 (2.8)	0.002	0.081

*Mann Whitney U-test for nonparametric variables*.

* *General linear model, multivariate, with General Ability. Bonferroni correction for multiple comparisons: significant p-values (p < 0.001) in bold*.

*MCI, Mild Cognitive Impairment, CVLT-II, California Verbal Learning Test 2rd edition, WAIS-IV, Wechsler Adult Intelligence Scale 4th edition, WMS-III, Wechsler Memory Scale 3rd edition, D-KEFS. Delis Kaplan Executive Function System*.

### Z-score comparison

**Figure 2:**
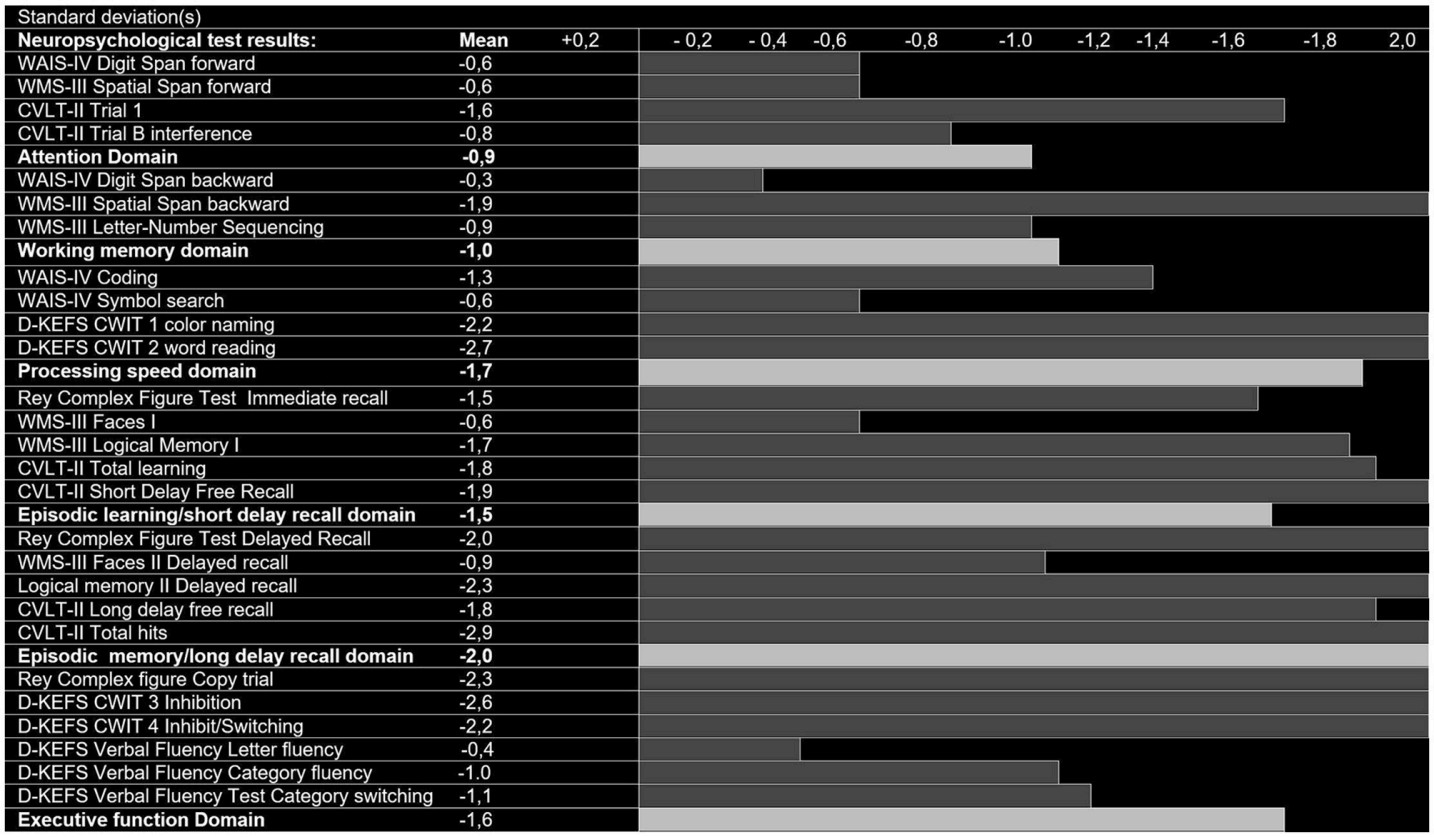
Neuropsychological test results (SD below zero), control group-derived z-scores in patients with amnestic Mild Cognitive Impairment (aMCI, *n* = 35).

**Figure 3:**
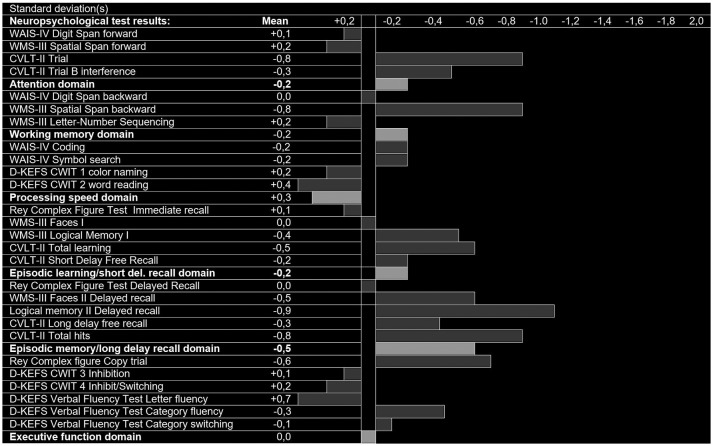
Neuropsychological test results (SD below zero), control group-derived z-scores in patients with nonamnestic Mild Cognitive Impairment (naMCI, *n* = 16).

Figures 2, 3 show the z-scores of the MCI patients on all the neuropsychological measures with controls as reference, on a scale that ranges from + 0.4 standard deviations (z-score = 0.4) to – 2.0 standard deviations (z-score = −2) from the mean, in addition to the domain scores. Figures 2, 3 shows the results of the amnestic and nonamnestic MCI subtype, respectively. The amnestic MCI subtype results displayed reduced scores (below zero) as compared to the control group on every neuropsychological measure and for all cognitive domains, and several measures were 2 standard deviations below the mean of the control group. For the nonamnestic MCI subtype, some of the results were on the positive side of zero ranging from +0.4 to −0.9 standard deviations from the mean of the controls. Only the domains scores were used in the structure-function analyses.

The amnestic MCI subtype results displayed reduced scores (below zero) as compared to the control group on every neuropsychological measure and for all cognitive domains, and several measures were two standard deviations below the mean of the control group (Figure 2). For the nonamnestic MCI subtype, some of the results were on the positive side of zero ranging from +0.4 to −0.9 standard deviations from the mean of the controls (Figure 3).

### Neuroimaging results

Table 7 presents the number of persons with pathological scores on the different MRI scoring systems. Thirty one individuals (61%) in the MCI group and 17 (33%) in the control group had at least one pathological neuroimaging score (*p* = 0.010), and 17 (33%) MCI patients and 7 (14%) controls had pathological results on more than one neuroimaging scale (*p* = 0.057). Hippocampal atrophy, measured by a pathological MTA score, according to the age cut-off, were found in 24 of the participants; 19 (54%) in the amnestic MCI group, two (12, 5%) in the nonamnestic MCI group and three (6%) in the control group. The MTA score differed significantly when comparing the MCI group with controls, when using two tailed independent *t*-test (*p* < 0.0001), and when comparing the amnestic MCI and nonamnestic MCI group (*p* = 0.006). A pathological PA score was found in 23 subjects; nine (26%) in the amnestic group, four (25%) in the nonamnestic group and 10 (20%) in the control group (*p* = 0.477). Similarly, Fazekas score was rated as pathological for 23 subjects, nine (26%) in the amnestic MCI group, four (25%) in the nonamnestic MCI group, and 10 (20%) in the control group (*p* = 0.477). A total of 11 participants had pathological GCA-F scores; seven (20%) in the amnestic subgroup, none in the nonamnestic subgroup and four (7%) in the control group. The mean GCA-F score was not different between MCI patients and controls, but there was a significant mean group difference (*p* = 0.002) between the amnestic and the nonamnestic MCI groups.

**Table 7:** Prevalence of pathological MRI scores and their statistical characteristics according to diagnosis.

**MRI scoring systems/prevalence:**	**Controls *n* = 51**	**MCI *n* = 51**	***p*-value**	**aMCI *n* = 35**	**naMCI *n* = 16**	***p*-value**
MTA (%)	3(6%)	21(41%)	***0.000***	19(54%)	2 (12,5%)	***0.006***
PA (%)	10(20%)	13(25%)	*0.477*	9(26%)	4(25%)	*0.957*
GCA-F (%)	4(7%)	7(14%)	*0.338*	7(20%)	0(0%)	*0.054*
Fazekas Dicom (SD) (%)	10(20%)	13(25%)	0.477	9(26%)	4(25%)	0.957
# N with pathological score (%)	17(33%)	31(61%)	***0.010***	25(71%)	6(37.5%)	*0.031*
# N with ≥ 2 pathological scores (%)	7(13%)	16(31%)	*0.057*	13(37%)	3(18%)	*0.329*
**MRI SCORING SYSTEMS/CHAR:**						
MTA combined mean (SD)	0.314(0.469)	0.902(0.860)	***0.000***	1.04(0.915)	0.594(0.66)	*0.054*
PA mean(SD)	0.94(0.732)	1.04(0.732)	*0.776*	0.97(0.568)	0.69(0.704)	*0.776*
GCA-F mean (SD)	0.71(610)	0.75(0.688)	*0.761*	0.91(0.702)	0.38(0.500)	***0.002***
Fazekas (SD)	1.14(0.722)	1.23(0.690)	*0.398*	0.94(0.772)	1.02(0.678)	*0.184*

*Prevalence group difference significance by Chi Square, Mean difference by two tailed independent sample T-test*.

*MCI, Mild cognitive impairment, aMCI, amnestic MCI, naMCI, nonamnestic MCI, MTA, Medial temporal lobe atrophy score; GCA-F, Global cortical atrophy score—Frontal subscore; PA, Koedam score for parietal atrophy. Significant p-values (p < 0.05) in bold*.

### Structure—function relationships

Table 8 shows the relationships between cognitive domain z-scores and the different MRI scores in the MCI group. The MTA score showed a significant correlation with episodic memory/long delay recall domain score (*R*^2^ = 0.100, *p* = 0.043). PA score significantly correlated with working memory domain scores (*R*^2^ = 0.106, *p* = 0.043), while GCA-F score significantly correlated with episodic learning/short delay recall domain scores (*R*^2^ = 0.100, *p* = 0.036). An increased radiological atrophy score correlated to lower performance score. Fazekas score showed no significant correlation with either of the cognitive domain scores. When looking at structure-function relationships in the control group, no correlations were found between MRI scores and domain scores, except between the GCA score and processing speed (*p* = 0.006). However, only four controls obtained pathological GCA scores.

**Table 8:** Correlations between MRI scores and cognitive domains Z-scores in MCI patients.

**Domain name/ radiological score**		**MTA**	**PA**	**Fazekas**	**GCA-F**
Attention domain	*p(CI95)*	*0.565*(−0.319–0.176)	*0.231*(−0.455–0.087)	*0.377*(−0.477–0.172)	*0.159*(−0.553–0.093)
	*R*2	0.019	0.042	0.029	0.053
Processing speed domain	*p(CI95)*	*0.157*(−0.757– 0.126)	*0.778*(−0.450–0.597)	*0.573*(−0.725–0.406)	*0.094*(−0.088–1.076)
	*R*2	0.088	0.050	0.012	0.104
Working memory domain	*p(CI95)*	*0.139*(−0.521–0.075)	***0.043*****(**–**0.690–** – **0.011)**	0.759(−0.443–0.325)	*0.093*(−0.729–0.059)
	*R*2	0.069	**0.106**	0.026	0.081
Episodic learning/short delay recall domain	*p(CI95)*	*0.105*(−0.623–0.061)	*0.247*(−0.637–0.168)	*0.832*(−0.489–0.395)	***0.036*****(**–**0.924–** – **0.031)**
	*R*2	0.065	0.039	0.005	**0.100**
Episodic memory/long delay recall domain	*p(CI95)*	***0.043*****(**–**0.706**–−**0.011)**	*0.355(*–*0.612–0.224)*	*0.659*(−0.556–0.335)	*0.072*(−0.896–0.040)
	*R*2	0.**100**	0.035	0.022	0.084
Executive function domain	*p(CI95)*	*0.876*(−0.113–0.132)	*0.224*(−0.226–0.054)	*0.787*(−0.175–0.133)	0.*662*(−0.127–0.199)
	*R*2	0.006	0.036	0.007	0.009

*MTA, Scheltens Middle temporal lobe atrophy score, Fazekas White matter hyperintensity score. (Fazekas score). Average Frontal subscore of Global Cortical Atrophy score (GCA-F). Posterior atrophy score (PA). CI(95%) for Beta. Domain Z scores are composed from a mean score from the respective domain scores. Linear regression analyses were performed with age and sex as covariates. Significant p-values (p < 0.05) in bold*.

## Discussion

The study presents descriptive cross-sectional data on functional and structural profiles of Memory clinics patients with MCI. As expected, patients diagnosed with MCI had overall lower performance on the neuropsychological tests and higher scores on visually rated MRI pathology scales compared to age, gender and SES-matched controls. Interestingly, after controlling for intelligence assessed by GAI from the test WAIS-IV, fewer of the neuropsychological tests remained abnormal in amnestic and nonamnestic MCI patients. One possible interpretation of this finding is that intelligence is a moderator of neuropsychological performance. Another interpretation is that intelligence is a confounder. One might argue that GAI is not a valid proxy of cognitive reserve in an MCI population, as the results of the tests that are included in the GAI may be reduced due to a disease process. It is one standard deviation (SD) difference between the MCI group and the control group. Nevertheless, the mean of all the individual subtests in the GAI in the MCI group lies within a normal range.

Apparently, intelligence operationalized as GAI moderates group differences on the cognitive profiles in patients with MCI compared to controls. GAI also moderates the difference on the profiles between patients with amnestic and nonamnestic MCI. These findings are consistent with the literature that emphasized low test scores do not equal pathology—obtaining multiple low scores may or may not be a sign of a pathological condition. Brooks and Iverson (2010) recommend utilizing knowledge of the frequency of low test scores (base rates) to interpret reduced performance in neuropsychological assessment in general. The base rates of low scores vary in relation to intelligence (Ingraham and Aiken, 1996; Crawford et al., 2007; Brooks and Iverson, 2010; Smith and Bondi, 2013). Without a robust operationalization of, or correction for, general cognitive function, too much emphasis may be placed on low scores for those with low general cognitive function, and similarly, too little on reduced test scores for those with high general cognitive function. Having one or more scores 1,5 SD below the mean is uncommon in cognitively healthy people with superior intelligence, and common in cognitively healthy people with lower intelligence (Brooks et al., 2007). If a clinician applies the same cut-off score for patients with either low or high general cognitive ability, this may lead to an underestimation of the cognitive deficits as “normal” in a high cognitively functioning person, or an overestimation of the cognitive deficits as “not normal” in a low cognitively functioning person. This might apply to commonly used cut-off scores for identifying MCI. One interpretation of our data is that it might be useful to control for intelligence by the use of GAI from WAIS-IV, in the interpretation of test results of patient suspected of having MCI. This might help minimize the risk of false positive diagnosis in individuals with low premorbid general abilities, and false negative diagnosis in individuals with high premorbid general abilities.

We found that individuals with amnestic MCI had significantly overall lower neuropsychological test performance compared to controls, with cognitive profiles that indicate severe functional impairment. They also had lower overall performance compared to the nonamnestic MCI subtype. This is in accordance with the large body of existing research on MCI, viewing amnestic MCI as a more severe pathological condition that diverges from cognitive changes associated with normal aging (Petersen et al., 1997; Petersen and Morris, 2005; Smith and Bondi, 2013).

As hypothesized, we found that the MCI group had higher MTA scores on the visual MRI scales compared to controls, which indicate higher prevalence of brain pathology in this patient group. The MCI group displayed significantly higher MTA scores than the control group. No significant difference was found between the groups when comparing the other visual scores. This is in accordance with Duara et al. (2013) and Rhodius-Meester et al. (2017) who reported MTA as the only scale that differentiated MCI patients from controls. In the present study, MTA scores also differentiated amnestic and nonamnestic MCI patients, but not nonamnestic MCI patients from controls. These results are consistent with findings of larger hippocampal volumes in nonamnestic MCI subtypes compared to amnestic subtypes, as reported by Vos et al. (2013) and van de Pol et al. (2009). However, our findings are contrary to studies reporting greater MTA scores in the nonamnestic MCI subtypes compared to controls (van de Pol et al., 2009; Vos et al., 2013). These different results may partly be explained by a higher average age of the patients with nonamnestic MCI subtypes compared to controls in previous studies. MTA score is considered age sensitive (Rhodius-Meester et al., 2017) and more frequently present in individuals older than 70 years of age (van de Pol et al., 2009).

GCA-F scores indicated greater frontal lobe atrophy in the amnestic MCI subtype compared to the nonamnestic subtype. Whitwell et al. (2008) showed regional frontal atrophy in both the amnestic multiple domain MCI group and the single domain nonamnestic MCI group by using an automated segmentation method. None of our nonamnestic participants had high GCA-F scores. One explanation might be that the atrophy is more localized in these individuals and therefore not severe enough to be identified by the GCA-F scale. Our sample size of nonamnestic patients is small, and the results may diverge in a larger study sample.

The PA rating showed similar mean group scores between the amnestic MCI and nonamnestic MCI subtype groups. This finding is consistent with previous volumetric studies that found no difference in parietal lobe volumes between subtypes of MCI patients (Whitwell et al., 2008; van de Pol et al., 2009). Similarly, the Fazekas scale ratings did not differentiate the subtype groups in the present study. Previous studies of MCI patients have found a stronger association to age than MCI subtypes using the Fazekas scale (Bombois et al., 2007; Rhodius-Meester et al., 2017). Hence, the lack of group differences in pathological Fazekas score between MCI patients and controls in the present study is in agreement with previous population studies (Schmidt et al., 2011; Prins and Scheltens, 2015; Claus et al., 2016). The MCI-patients with higher MTA scores had the greatest reduction in performance on tests related to episodic memory. This finding is in line with the large body of prior studies demonstrating that the hippocampus is one of the neural substrates for episodic memory formation (Ranganath et al., 2005; Nichols et al., 2006; Lewis-Peacock and Postle, 2008).

In contrast, the frontal lobe score GCA-F correlated with performance on the episodic learning. This is in accordance with studies reporting that episodic learning (encoding) is mediated by brain structures involving the prefrontal cortex in addition to hippocampus (Nee and Jonides, 2011; Harding et al., 2015). Although the frontal lobes is known to be involved in executive function, the frontal lobe score GCA-F did not correlate with performance on the executive functioning domain scores in the MCI patients in our study. The problems with operationalizing executive functions has been addressed previously in a meta-analysis by Alvarez and Emory (2006), were they examine the validity of the executive function-construct as measured by cognitive tests in relation to frontal lobe damage. They concluded with “inconsistent support for the historical association between executive functions and the frontal lobes” (Alvarez and Emory, 2006, p. 33). Recent studies has focused on executive functions in relation to neural networks, rather than a regional anatomical/structural frame of reference (Weiler et al., 2014; Beaty et al., 2015; Crittenden et al., 2015; Brown et al., 2017; Filippi et al., 2017). The lack of correlation between the GCA-F scores and the executive function domain may also be due to the fact that some executive function tests have high sensitivity for assessing brain injury, but may have low specificity. Damage to a wide variety of brain regions may affect executive function test performance while isolated frontal damage may not always result in deficits in executive function that can be detected by tests (Mesulam, 1998; Strauss et al., 2006; Hestad and Egeland, 2010; Lezak, 2013). Furthermore, in our MCI patients, the scores on the working memory domain correlated inversely with the parietal lobe atrophy score. This finding is similar to a previous study that found decreased connectivity between the prefrontal cortex and posterior parietal regions in early onset AD (Filippi et al., 2017). Atrophy of parietal regions is also correlated with reduced working memory function in functional MRI (fMRI) studies of healthy controls (Honey et al., 2002). PA score on structural MRI may be a useful indicator of reduced working memory function in MCI patients. Since the PA score may be more readily used in clinical settings than fMRI and semi-automated morphometric analyses, it may be a convenient tool for clinicians as an objective biomarker to corroborate with neuropsychological assessment of the patients' working memory function. The lack of correlation between the Fazekas score and any neuropsychological domain scores in our MCI patients may be a result of the minimal regional information included in the Fazekas rating scale. The literature regarding WMH and their relationship to neuropsychological domains remains somewhat controversial. Some studies have reported that a greater white matter hyperintensity frequency is associated with poorer executive function and/or slower processing speed (Tullberg et al., 2004; Prins and Scheltens, 2015). Other studies have reported a lack of domain-specific relationship, but found an impact on global cognition (Overdorp et al., 2014).

## Strengths and limitations

A strength of this study is that the study sample is well-defined, consisting of patients diagnosed in hospital-based Memory clinics by experienced multidisciplinary teams using national assessment guidelines. Furthermore, we matched the groups on SES in addition to age and sex. We used three scales for the brain MRI measures that took in to consideration both neurodegenerative and vascular factors. We included comprehensive neuropsychological assessment in order to cover all major neuropsychological domains, administrated by the same experienced neuropsychologist. The MRI scoring was performed by two experienced radiologists blinded to the group adherence and viewing the images independently and had consensus ratings when discrepancies occurred. A limitation is that the study population is small; therefore the results should be interpreted with caution. As such, nonsignificant group differences may be due to insufficient power from the relatively small sample size. Low specificity and high sensitivity of the neuropsychological tests may have impacted the lack of correlation between the GCA-F and the executive function domain tests.

## Conclusion and clinical applications

Intelligence emerges as a strong covariate in the analyses of group differences in the cognitive profiles. Based on our data, we consider GAI useful as an operationalization of the general cognitive function criteria of MCI. Further, applying the GAI in general clinical assessments of MCI patients may be helpful in the diagnostic process to reduce the risk of false positive or false negative diagnosis by relating the neuropsychological test results to each individual's GAI-result before confirming the diagnosis of MCI.

The tests less influenced by GAI in our study were the tasks within the verbal episodic learning and memory domain, and a verbal fluency (categorizing) task within the executive function domain. Patients with the amnestic MCI subtype was expected to have poorer cognitive outcomes. In this material their neuropsychological profiles emerged as significantly impaired in multiple cognitive domains compared to the nonamnestic MCI patients.

Our findings may suggest that neuropsychological tests and the MRI rating scores measure different aspects of the MCI condition. Also, patients with MCI is a heterogeneous group that have a variety of reasons for their cognitive impairment, and the impairment do not necessarily have a structural brain correlate. However, the usefulness of the MRI rating scores, except for the MTA scores, appears to be low in identifying an MCI condition. In older adults with MCI, a pathological MTA score suggests that the patient should be further assessed for MCI. However, a MTA score within the normal range does not exclude MCI.

## Ethics statement

This study was carried out in accordance with the recommendations of the Norwegian Regional Committee for medical and health research ethics, South-Eastern Health region (no: 2013/410) and by the Department of Research at each collaborating hospital with written informed consent from all subjects. All subjects gave written informed consent in accordance with the Declaration of Helsinki. The protocol was approved by the Norwegian Regional Committee for medical and health research ethics, South-Eastern Health region (no: 2013/410).

## Author contributions

MF conception and design of the study's neuropsychological part, data collection, data analysis and interpretation, drafting the article, critical revision of the article and final approval of the version to be published. HH conception and design of the study's radiological part, data collection, data analysis and interpretation, drafting the article, critical revision of the article and final approval of the version to be published. SH and JS conception and design of the study, data collection, data analysis and interpretation, drafting the article, critical revision of the article and final approval of the version to be published. LC conception and design of the study, data interpretation, critical revision of the article and final approval of the version to be published. TE conception and design of the study's radiological part and final approval of the version to be published. AE, B-OM, A-BK, and TL data collection and critical revision of the article and final approval of the version to be published. KB data analysis, critical revision of the article and final approval of the version to be published. EL data analysis and interpretation of the study's radiological part, critical revision of the article and final approval of the version to be published. IU input on the study design, data collection and critical revision of the article and final approval of the version to be published. GL P.I of the study, conception and design of the study, data collection, data analysis and interpretation, drafting the article, critical revision of the article and final approval of the version to be published.

### Conflict of interest statement

The authors declare that the research was conducted in the absence of any commercial or financial relationships that could be construed as a potential conflict of interest.
