# Corrigendum: Cognitive Profiles and Atrophy Ratings on MRI in Senior Patients With Mild Cognitive Impairment

**DOI:** 10.3389/fnagi.2019.00037

**Published:** 2019-02-26

**Authors:** Marianne M. Flak, Haakon R. Hol, Susanne S. Hernes, Linda Chang, Thomas Ernst, Andreas Engvig, Knut Jørgen Bjuland, Bengt-Ove Madsen, Elisabeth M. S. Lindland, Anne-Brita Knapskog, Ingun D. Ulstein, Trine E. E. Lona, Jon Skranes, Gro C. C. Løhaugen

**Affiliations:** ^1^Department of Clinical and Molecular Medicine, Norwegian University of Science and Technology, Trondheim, Norway; ^2^Department of Pediatrics, Sørlandet Hospital HF, Arendal, Norway; ^3^Department of Radiology, Sørlandet Hospital HF, Arendal, Norway; ^4^Department of Clinical Science, University of Bergen, Bergen, Norway; ^5^The Memory Clinic Geriatric Unit, Department of Medicine, Sørlandet Hospital, Arendal, Norway; ^6^Department of Diagnostic Radiology and Nuclear Medicine, and Department of Neurology, University of Maryland School of Medicine, Baltimore, MD, United States; ^7^Department of Neurology, Johns Hopkins University School of Medicine, Baltimore, MD, United States; ^8^Department of Medicine, John A. Burns School of Medicine, University of Hawaii at Manoa, Honolulu, HI, United States; ^9^Department of Medicine, Diakonhjemmet Hospital, Oslo, Norway; ^10^Department of Research, Sørlandet Hospital, Arendal, Norway; ^11^Department of Radiology and Nuclear Medicine, Oslo University Hospital, Oslo, Norway; ^12^Institute of Clinical Medicine, University of Oslo, Oslo, Norway; ^13^Department of Geriatric Medicine, The Memory Clinic, Oslo University Hospital, Oslo, Norway; ^14^Department of Psychiatry, Age Psychiatry, The Hospital of Telemark, Skien, Norway

**Keywords:** MCI, intelligence, memory clinic patients, cognitive dysfunction, brain pathology, structural magnetic resonance imaging, neuropsychological functioning, neuropsychological tests

In the published article, there was an error in the affiliation order and numbering. The corrected affiliation order and numbering should be as follows:

“Marianne M. Flak^1,2^^*^, Haakon R. Hol^3,4^, Susanne S. Hernes^4,5^, Linda Chang^6,7,8^, Thomas Ernst^6,7,8^, Andreas Engvig^9^, Knut Jørgen Bjuland^10^, Bengt-Ove Madsen^5^, Elisabeth M. S. Lindland^3,11,12^, Anne-Brita Knapskog^13^, Ingun D. Ulstein^13^, Trine E. E. Lona^14^, Jon Skranes^1,2^ and Gro C. C. Løhaugen^2^”

There was also an error regarding the affiliations for Professor Linda Chang. As well as having affiliation 6, they should also have “Department of Diagnostic Radiology and Nuclear Medicine, and Department of Neurology, University of Maryland School of Medicine, Baltimore, MD, United States” and “Department of Neurology, Johns Hopkins University School of Medicine, Baltimore, MD, United States”

Additionally, there was also an error regarding the affiliation of Professor Thomas Ernst. As well as having affiliation 6, they should also have “Department of Diagnostic Radiology and Nuclear Medicine, and Department of Neurology, University of Maryland School of Medicine, Baltimore, MD, United States” and “Department of Neurology, Johns Hopkins University School of Medicine, Baltimore, MD, United States”

The authors apologize for this error and state that this does not change the scientific conclusions of the article in any way. The original article has been updated.

